# Gut Microbiome Succession in Chinese Mitten Crab *Eriocheir sinensis* During Seawater–Freshwater Migration

**DOI:** 10.3389/fmicb.2022.858508

**Published:** 2022-03-30

**Authors:** Chenxi Shao, Wenqian Zhao, Nannan Li, Yinkang Li, Huiming Zhang, Jingjing Li, Zhiqiang Xu, Jianjun Wang, Tianheng Gao

**Affiliations:** ^1^Department of Marine Biology, College of Oceanography, Hohai University, Nanjing, China; ^2^State Key Laboratory of Lake Science and Environment, Nanjing Institute of Geography and Limnology, Chinese Academy of Sciences, Nanjing, China; ^3^Freshwater Fisheries Research Institute of Jiangsu Province, Nanjing, China

**Keywords:** gut microbiome succession during migration gut microbiome, Chinese mitten crab, salinity, migration, seawater–freshwater transition

## Abstract

Biological migration is usually associated with disturbances and environmental changes that are key drivers in determining the diversity, community compositions, and function of gut microbiome. However, little is known about how gut microbiome is affected by disturbance such as salinity changes during migration from seawater to freshwater. Here, we tracked the gut microbiome succession of Chinese mitten crabs (*Eriocheir sinensis*) during their migrations from seawater to freshwater and afterward using 16S rDNA sequencing for 127 days, and explored the temporal patterns in microbial diversity and the underlying environmental factors. The species richness of gut microbiome showed a hump-shaped trend over time during seawater–freshwater migration. The community dissimilarities of gut microbiome increased significantly with day change. The turnover rate of gut microbiome community was higher during seawater–freshwater transition (1–5 days) than that in later freshwater conditions. Salinity was the major factor leading to the alpha diversity and community dissimilarity of gut microbiome during seawater–freshwater transition, while the host selection showed dominant effects during freshwater stage. The transitivity, connectivity, and average clustering coefficient of gut microbial co-occurrence networks showed decreased trends, while modularity increased during seawater–freshwater migration. For metabolic pathways, “Amino Acid Metabolism” and “Lipid Metabolism” were higher during seawater–freshwater transition than in freshwater. This study advances our mechanistic understanding of the assembly and succession of gut microbiota, which provides new insights into the gut ecology of other aquatic animals.

## Highlights

- Crab gut microbiome showed a hump-shaped pattern in species richness during seawater–freshwater migration.
- The turnover rate of gut microbiome was higher during seawater–freshwater transition than in freshwater.

- Salinity primarily affected the diversity and community compositions of gut microbiome during seawater–freshwater transition.
- The transitivity, connectivity, and average clustering coefficient of gut microbial co-occurrence networks showed decreased trends, while modularity increased during seawater–freshwater migration.
- “Amino Acid Metabolism” and “Lipid Metabolism” were higher during seawater-freshwater transition than freshwater.

## Introduction

Migration as a form of ecological succession has been an important theme of great concern involved in ecology for lots of years. However, the machine underlying gut microbiota succession in aquatic animals remains unidentifiable (Wang et al., 2019), especially in crustaceans during migration. The gut microbiome could be affected by host phylogeny and exogenous changes (Wong and Rawls, 2012). For instance, environmental factors such as habitat (Lai et al., 2020) and salinity (Lai et al., 2020) can affect gut microbiota composition and species diversity. The gut microbiome as a “filter” is restricted by ambient environments (Yan et al., 2016). Theoretically, all microbiomes colonize in the aquatic organism gut are expected to be derived from surrounding water environments. The findings on gut microbiome of migratory habit of gray mullet show that the gut microbiome of adult fish migrating to different geographical tracts harbor and their respective migration routes are similar to historical records of seawater microbiome (Le and Wang, 2020). In addition, the gut microbiome of medaka under different salinity stress regulates various pathways and facilitates the salinity acclimation of both the host and the bacteria, during seawater transfer freshwater (Lai et al., 2020). However, recent studies reveal that host selection due to genetics is a key factor to filtering gut microbiota (Xiao et al., 2021). Despite the strong influence of geographical factors on the migrating human gut microbiota, cultural similarity due to a shared ethnic origin drives the presence of a shared gut microbiota composition (Dwiyanto et al., 2021). The gut microbiome of swan geese or Atlantic salmon, while somewhat altered after migration, still maintains a core group of species (Dehler et al., 2017; Rudi et al., 2018; Wu et al., 2018). So far, only a few gut microbiome studies consider the host developmental issue, but gut microbiome diversity and composition could be significantly affected by the host development (Yan et al., 2016). Host development prevails over environmental dispersal in governing the ecological succession of zebrafish gut microbiota (Xiao et al., 2021). During migrations, we thus expect that the gut microbiome is associated with environmental changes and host selection, but what the most pivotal factors are is yet unknown in the Chinese mitten crab.

**FIGURE 1:**
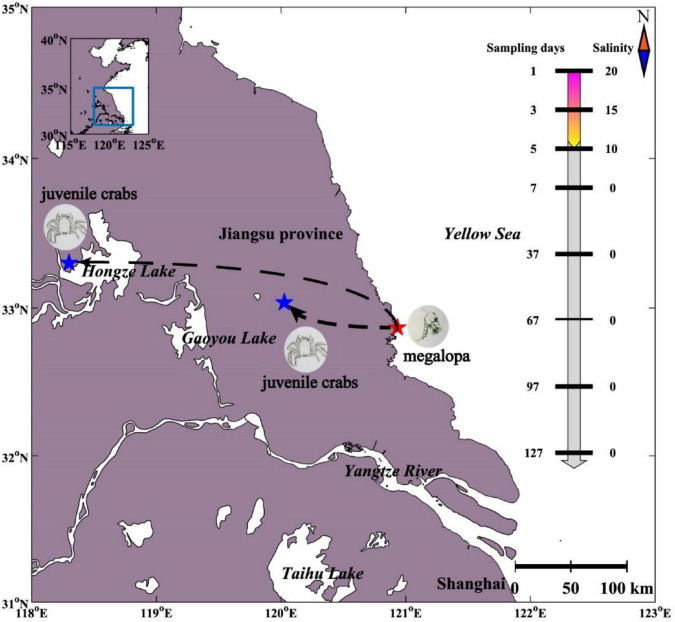
Experimental design of gut microbiome sampling of Chinese mitten crab. The sampling locations at different sampling days and salinity corresponding to each sampling day are shown.

The Chinese mitten crab (*Eriocheir sinensis*) is a euryhaline species with the characteristics of a catadromous life cycle with migrations between freshwater and seawater. For instance, the mature female adults hatch the eggs which metamorphose into megalopa and live in the seawater. The megalopa metamorphoses into juvenile crab usually in May, which then actively migrates upstream into freshwaters with a benthic life until sexual maturity (Figure 1; Anger, 1991; Lafontaine and Veilleux, 2007). The changes of the water environment during migration will inevitably lead to changes in the diversity and community compositions of gut microbiome, which as an organ that regulates osmotic pressure. Recently, gut microbiome has been shown to play an important role in host physiological functions, including growth (Yan et al., 2012), metabolism (Diaz Heijtz et al., 2011), and regulation of digestion (Stanley et al., 2012). Although extensive researches had been carried out for Chinese mitten crabs (Gao et al., 2018), little is known about the gut microbiome of Chinese mitten crabs especially during the seawater–freshwater migrations.

Here, we tracked the process of Chinese mitten crabs from seawater–freshwater migration and explored the changing patterns and driving factors of their gut microbiome using 16SrDNA sequencing. The Chinese mitten crab is an excellent species to study how the migration affects the assembly and succession of gut microbiota. During the migration process, Chinese mitten crabs constantly experience changes in environmental conditions, such as salinity, temperature, and pressure, and may cause stress to host and associated gut microbiome. We had the following three objectives: (1) How do the biodiversity and community compositions of the Chinese mitten crab gut microbiota change during seawater–freshwater migration? (2) What is the main factor that drives gut microbiome shift during migration? (3) How do the co-occurrence patterns and function of gut microbiome change during migration?

## Materials and Methods

### Experimental Design and Sample Collection

The gut of Chinese mitten crab (*Eriocheir sinensis*) larvae (*n* = 9) were collected from a seawater breeding pond in Dongtai City (DT) (32.868°N, 120.933°E) on the east coast of China in May 2020 (Figure 1). During seawater–freshwater transition (1–5 days), one-fourth of the water was replaced every day with freshwater water every day in a breeding pond, and the salinity of the pond water dropped from 20 PSU (practical salinity units) to 10 PSU within 5 days. We collected intestinal samples on the first (*n* = 3, 20 PSU), the third (*n* = 3, 15 PSU), and the fifth day (*n* = 3, 10 PSU) separately. After the fifth day, the Chinese mitten crab larvae in the breeding pond were transported to the freshwater breeding ponds in Sihong (SH) (33.301°N, 118.300°E) and Xinghua (XH) (33.036°N, 120.026°E) (0 PSU). In freshwater, the intestinal samples were collected every 30 days (*n* = 6) from May 7, 2021, to September 7, 2021, (*n* = 30). For larval samples, sterile water was used to rinse the surface and remove the intestines through a dissecting needle. For crab samples, the surface of the crab was washed with sterilized water and 75% alcohol and then left for 3–5 min at room temperature. The contents of the hind intestine were collected using a scalpel. The samples were stored in sterile tubes under −80^°^C until further molecular analyses. In addition, four environmental variables were measured or collected (Supplementary Table 1). Water temperature (WT) and salinity were recorded *in situ* at a depth of 10 cm (below the water surface) using corresponding probes (Oxi 340i; WTW, Weilheim, Germany). The concentrations of water phosphate (PO_4_^3−^) and ammonia nitrogen (NH_3_^+^) were analyzed according to the seawater analysis standard of China (AQSIQ, 2007).

### Community Analyses

Bacterial 16S rDNA amplicon analysis was performed on all intestinal samples (*n* = 39) by Illumina sequencing. Due to the insufficient amount of DNA obtained from the intestine of a single crab, every three intestines were pooled together to compose one biological sample for each pond. DNA was extracted using OMEGA kit E.Z.N. A™ Mag-Bind Soil DNA Kit according to manufacturer’s instructions and was verified by 0.8% agarose gel electrophoresis. Then V3 and V4 hypervariable regions of prokaryotic 16S rDNA were amplified to generate amplicons, and the forward primers containing the sequence 341F 5′-CCTACGGGNGGCWGCAG-3′ and the reverse primers containing the sequence 805R 5′-GACTACHVGGGTATCTAATCC-3′ were used for classification analysis. For each sample, triplicate 30-μl PCRs were performed, which contained 20 ng DNA extracts as a template with the following conditions: 25 cycles of denaturation at 95°C for 30 s, annealing at 55°C for 30 s, followed by 5 three-step cycles of 20 s at 94°C, 20 s at 55°C, and 30 s and 72°C, and one final fold at 72°C for 5 min. All PCR amplicons were detected by 2% agarose gel electrophoresis, and the library concentration was determined using Qubit3.0 (Invitrogen, United States) fluorescence quantifier. The Prep Kit (Illumina, San Diego, CA, United States) was used according to the manufacturer’s instruction, and 300-bp pair-ended insertion was sequenced using MiSeq Reagent Kit V3 chemistry on Illumina MiSeq platform owned by Sangon Biotech Co., Ltd. (Shanghai, China).

Further, the 16S rDNA amplicon raw reads were processed according to the previous literatures (Zhang et al., 2018). The paired-end reads were merged and relabeled using “-fastq_mergepairs”; reads with low-quality (error rates > 0.01) and redundancy were filtered and dereplicated using “-fastq_filter” and “derep_fulllength,” respectively. The *de novo* biological sequences, ASVs (exact sequence variants), were clustered, and chimeras were filtered using “-unoise3.” Subsequently, the operational taxonomic units (OTUs) table was created by “-otutab.” Through the ribosome database (RDP) Bayesian algorithm (Wang et al., 2007), the OTU sequence was classified with a threshold of 0.6 and the species annotations were divided into five levels: phylum, class, order, family, and genus; and finally an OTU feature table was generated. All archaeal, chloroplast, and mitochondrial sequences have been deleted, as well as the other sequences that cannot be assigned to bacteria.

### Statistical Analyses

Diversity is a composite quantity composed of richness and evenness components (Lou, 2010), Pielou’s evenness [*J* = H/log (S), where H is the Shannon-Weaver diversity index and S is the number of species] (Pielou, 1967) is a good measure of the distribution of relative abundance in a community. We chose species richness and Pielou’s evenness as biodiversity metrics reflecting the two aspects of community biodiversity. For community composition, considering that the species occurrence percentages were larger than 85%, we only included the highly abundant bacterial phyla. The relationships between richness and elevation, evenness and elevation, and relative abundance of phyla were explored with linear and quadratic models. The most appropriate model was selected based on the lowest value of Akaike’s information criterion (Yamaoka et al., 1978).

The variation in bacterial beta diversity uses the matrices based on Bray-Curtis dissimilarity to examine the dissimilarity in community composition. The distance of sampling day was measured as the Euclidean distance with a log10 transformation. Then, the variations in bacterial beta diversity were plotted against the changes in distance of sampling day. This distance decay relationship was analyzed using a Gaussian generalized linear model, and the significance was determined using Mantel tests (Pearson’s correlation) on 9,999 permutations. The turnover rate of bacterial communities was calculated as the slope of models. In addition, a non-metric multidimensional scaling (NMDS) analysis was used to compare the differences during seawater–freshwater transition and freshwater gut bacterial communities based on Bray-Curtis distances. We applied random forest model (Feld et al., 2016) and redundancy analysis (RDA) to identify the important factors affecting each OTU. Pearson’s correlation coefficients were used to evaluate the statistical correlation between the explanatory variables, and the variables with high correlation coefficients (Pearson *r* > 0.7) were excluded from the models.

To describe the complex co-occurrence patterns in gut microbiome networks, SparCC was used to construct the association network. We only retained more than 50% of OTUs present in all samples for network construction (Langfelder and Horvath, 2012). The nodes and the edges in the network represent OTUs and the correlations between pairs of OTUs, respectively. A set of network topological properties, such as transitivity (the probability that the adjacent nodes of a node are connected, also called the clustering coefficient) and degree (the number of adjacent edges), were calculated for each co-occurrence network with the “igraph” package in R (Ma et al., 2016). Finally, significance tests were performed through an analysis using Wilcoxon test to examine whether the differences among comparisons are significant.

In order to identify functional pathways based on 16S communities, PICRUSt2 (v.2.3.0) was used to normalize the non-rarefied amplicon data by 16S rRNA gene copy number and to infer metagenomic contents (Louca and Doebeli, 2018). The inferred metagenomic functions were assigned using the Kyoto Encyclopedia of Genes and Genomes database (KEGG; May 2020 Release) to obtain KEGG pathway names. STAMP v2.1.3 (Parks et al., 2014) was used to test statistically significant differences in the pathway contributions to the parent terms using Welch’s *t*-test, and was corrected for multiple-testing by Benjamini-Hochberg false discovery rate (FDR). Biologically interpretable phenotypes such as Stress Tolerant, Gram staining, and Forms Biofilms within each indicated community were predicted based on the Greengenes OTU table using BugBase (Cai et al., 2021).

## Results

### Patterns in Bacterial Diversity and Community Composition Along Sampling Day

**FIGURE 2:**
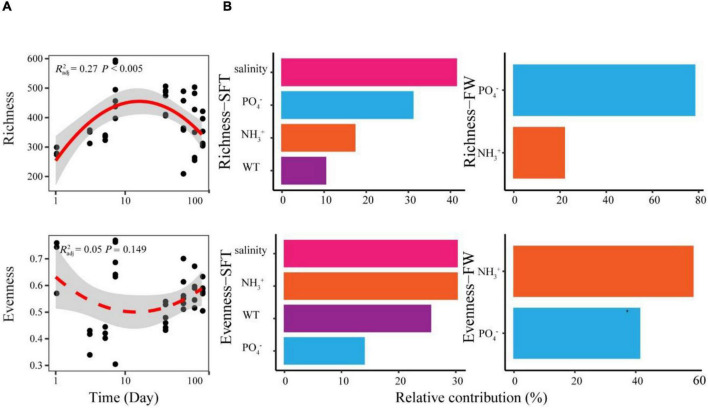
The temporal patterns and drivers of species richness and evenness of Chinese mitten crab gut microbiome during seawater–freshwater migration. **(A)** The relationships between days and alpha diversity were modeled with linear and quadratic models. The better model was selected based on the lower value of Akaike’s information criterion. The significant trend of the model is shown as solid line, and the non-significant trend is shown as dotted line. **(B)** The abiotic factors related to richness and evenness were identified with random forest during seawater–freshwater transition (SFT) and freshwater (FW), respectively. For abiotic factors, we considered phosphate (PO_4_^3−^), water temperature (WT), salinity, and ammonia nitrogen (NH${}_{3}^{+}$) of water.

We found significant relationships between species richness and sampling day of gut microbiome during seawater-freshwater migration (Figure 2A). More precisely, the species richness showed a hump-shaped pattern with the sampling day, showing the peak at 7 days. In addition, the species richness of gut microbiome during seawater–freshwater transition (1–5 days) was significantly higher than those in freshwater stage (Wilcoxon test, *p* < 0.05). However, the species evenness had no significant relationship with time (*p* = 0.103) (Figure 2A). The water salinity dropped from 20 to 10 PSU during the transition and remained at zero PSU in freshwater environments. Moreover, we found numerous environmental variables contributing to microbiome diversity during the two stages. The random forest analyses showed that salinity was the strongest variable correlated to the richness and evenness during seawater to freshwater transition (Figure 2B). Other environmental variables, such as PO_4_^3−^, temperature, and NH${}_{3}^{+}$ of water, also had important effects on richness and evenness during the seawater to freshwater transition (Figure 2B). However, the NH${}_{3}^{+}$ and PO_4_^3−^ were the most crucial factors for richness and evenness in the following freshwater stage, respectively.

**FIGURE 3:**
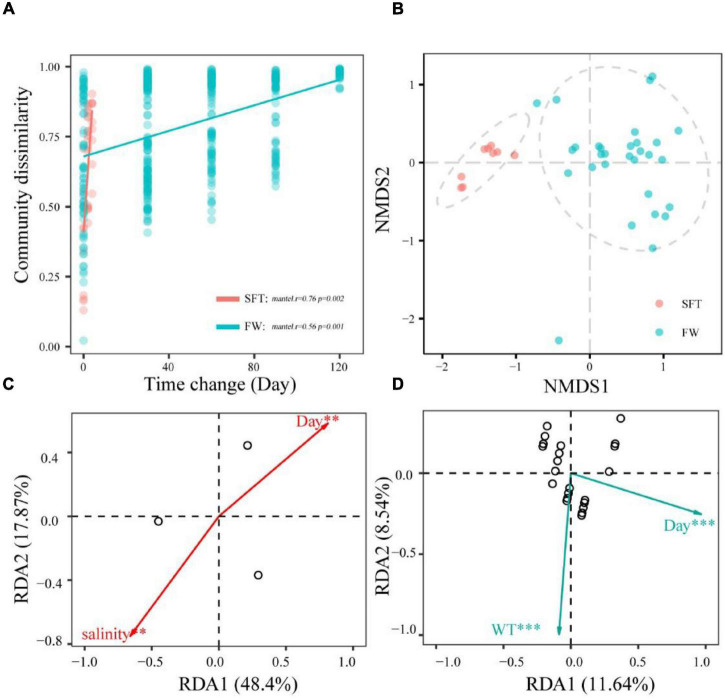
The relationships among the community dissimilarities and day change **(A)**; NMDS ordinations showing the structures of gut microbiome during seawater–freshwater transition (SFT) and freshwater (FW) **(B)**; the abiotic factors related to bacterial community **(C,D)**. The regression slopes of the linear relationships based on Gaussian generalized linear model are shown with solid lines. The relationships were statistically significant according to the Mantel test (9,999 permutations, *p* < 0.05) except for the bacterial communities. These factors were identified with redundancy analysis (RDA) during the seawater-freshwater transition (SFT) **(C)** and the freshwater stage (FW) **(D)**. For explanatory factors, we considered phosphate (PO_4_^3−^), water temperature (WT), salinity, and ammonia nitrogen (NH${}_{3}^{+}$) of water. Overlap among samples in panels **(C,D)** may due to smaller sample size. *p < 0.5; **p < 0.05; ***p < 0.01.

The community dissimilarity was positively (*p* < 0.01) related to the sampling day, showing significant distance-decay relationships with sampling day during seawater–freshwater transition (1–5 days) (*r* = 0.76, *p* < 0.01) and the freshwater stage (*r* = 0.56, p = 0.001) (Figure 3A). Notably, community turnover rates varied notably during migration, showing that the turnover rate in seawater (slope = 0.66) was significantly higher than that in freshwater (slope = 0.25). In addition, NMDS showed the gut microbiota of crab individuals of the same stages were generally grouped together (Figure 3B). Further, we found numerous variables regulating community dissimilarity in the two stages. For instance, salinity and sampling day were the important factors (*p* < 0.01) related to the community compositions in transition, while sampling day and WT showed significant correlations with community in freshwater (Figures 3C,D).

### Community Compositions in Gut Microbiome

**FIGURE 4:**
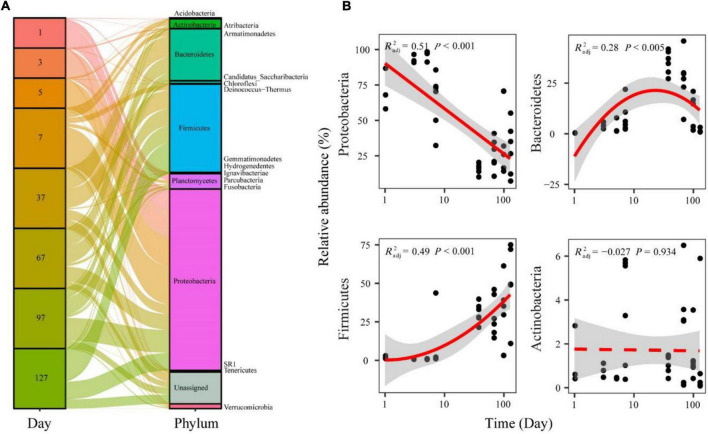
The relative abundance of bacterial phyla in the seawater–freshwater transition and freshwater stage **(A)**, and temporal patterns of dominant bacterial phyla **(B)**. The relationships between time and relative abundance were modeled with linear and quadratic models. The better model was selected based on the lower value of Akaike’s information criterion.

The represented OTUs were taxonomically assigned to 20 prokaryotic phyla (Supplementary Figure 1). During seawater to freshwater transition (1–5 days), the dominant abundant phylum was Proteobacteria (86.11%) (Figure 4A). In the later freshwater stage, the relatively abundant phyla were Proteobacteria (34.50%), Firmicutes (29.12%), and Bacteroidetes (16.57%) (Figure 4A). The relative abundance of main phyla all exhibited significant (*p* < 0.05) and temporal patterns. Specifically, Proteobacteria showed decreased trends over time during seawater–freshwater migration, while Firmicutes showed an increasing pattern (Figure 4B). In addition, the relative abundance of Bacteroidetes showed a hump-shaped trend over time (Figure 4B). We also found that the relative abundance of Proteobacteria in seawater–freshwater transition was significantly lower than those in freshwater (Wilcoxon test, *p* < 0.05), while the Firmicutes were higher (Wilcoxon test, *p* < 0.05).

### The Co-occurrence Network and Metabolic Functional Shifts of Gut Microbiome

**FIGURE 5:**
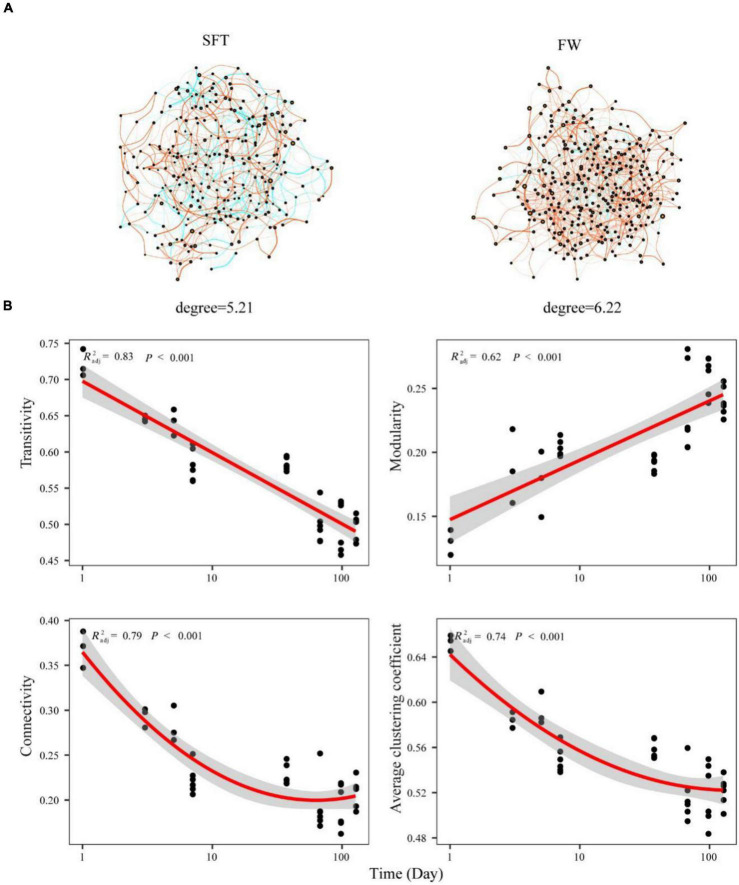
The co-occurrence network of gut microbiome on Chinese mitten crabs at the OTU level **(A)**. The nodes each represent unique OTUs in the data sets. The size of each node is proportional to the number of connections (that is, degree). OTUs are colored by different phyla. The green side is positive correlation and the red side is negative correlation. The relationships between network topological properties and time for gut microbiome community were modeled with linear and quadratic models **(B)**. The significant trend of the model is shown as solid line, and the non-significant trend is shown as dotted line. The better model was selected based on the lower value of Akaike’s information criterion.

To understand the role of biotic interaction in community assembly, the co-occurrence network of gut microbiome (at the OTU level) in Chinese mitten crabs was constructed based on the significant correlations during seawater–freshwater transition (1–5 days) and freshwater stage, respectively. Compared with the transition, OTUs were more densely linked in the network of the freshwater stage, with higher average degrees and more positive associations (Figure 5A). Some topological characteristics commonly used in network analysis were calculated to describe the complex interrelationship patterns between OTUs (Newman, 2003). Most categories of network topological characteristics of the crab gut microbiome exhibited obvious differences during seawater–freshwater migration (*p* < 0.05) (Figure 5B). The transitivity, connectivity, and average clustering coefficient exhibited decreased trends, while modularity increased over time during the seawater–freshwater migration (Figure 5B). In addition, the gut microbiome during seawater-freshwater transition had higher average clustering coefficient, connectivity, and transitivity than the freshwater stage (Wilcoxon test, *p* < 0.05).

**FIGURE 6:**
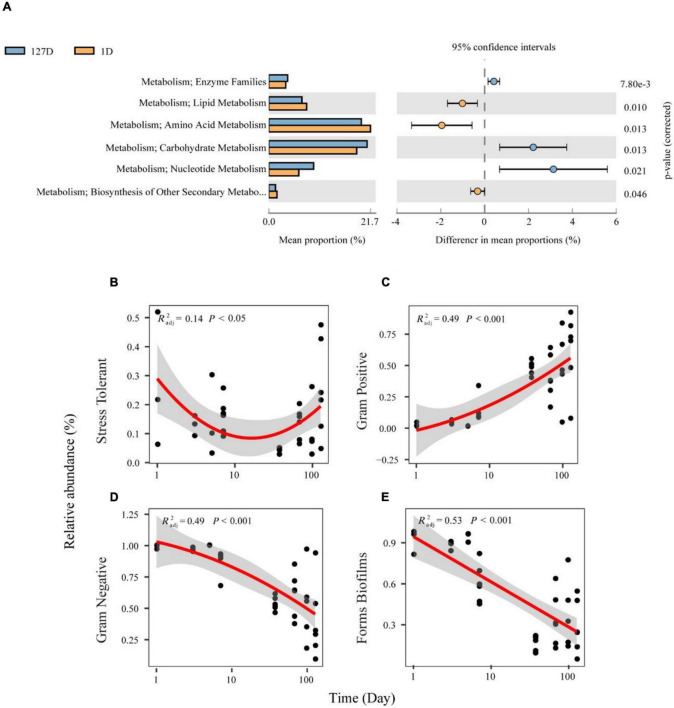
The functional profiles and organism level phenotypes of different microbial communities over time. Phylogenetic Investigation of Communities by Reconstruction of Unobserved States (PICRUSt) analysis was performed to identify the influenced biological pathways of the influence of salinity changes on the gut microbiome of the Chinese mitten crab **(A)**. Phenotype inference of bacterial communities from the Chinese mitten crab gut **(B–E)**. Relative abundances of bacteria differing in Gram staining are shown in panel **(C)** for Gram-negative and panel **(D)** for Gram-positive. Relative abundances of bacteria differing in latent pathogenicity phenotypes are shown in panel **(B)** for oxidative stress tolerance and panel **(E)** for biofilm formation.

We inferred the function and organism-level phenotypes of different microbial communities with PICRUSt and BugBase, respectively. There were 328 KEGG pathways from 8,398 ASVs present in the Chinese mitten crab gut microbiome, with an identity cutoff rate of 99%. The metabolism level 2 categories showed the different contributions during seawater–freshwater transition (1–5 days) and freshwater stage (*p* < 0.01, Welch’s *t*-test, Figure 6A). For example, “amino acid metabolism” and “lipid metabolism” had particularly a higher contribution during seawater-freshwater transition than in freshwater stage. However, “carbohydrate metabolism,” “enzyme families,” and “nucleotide metabolism” were conspicuously more abundant in freshwater (Figure 6A).

Further, the phenotypical characteristics of bacterial communities such as oxygen tolerance, Gram staining, and pathogenic potential were predicted (Supplementary Figure 2). Stress Tolerant and Gram-positive showed U-shaped and decreasing patterns over time (Figures 6B,C), respectively. On the contrary, Gram-positive and biofilms increased with time (Figures 6D,E). Compared with the transition stage, the gut microbiome in the freshwater stage has a significantly higher proportion of Gram-positive, while the proportion of biofilms formed by the gut microbiome was higher during transition (Wilcoxon test, *p* < 0.05).

## Discussion

Migration is a major area of interest within the field of ecology. Although extensive research has been carried out on migration (Dehler et al., 2017; Zhang et al., 2020), few studies have examined Chinese mitten crab gut microbiota during seawater-freshwater migrations. In this study, we explored the changes in the biodiversity, community compositions, and function of gut microbiome of Chinese mitten crabs in different periods of migration from seawater to freshwater. We found that (1) there were significant relationships between the species richness of gut microbiome and the sampling day, showing a hump-shaped pattern. (2) The community dissimilarities increased significantly over time during the seawater–freshwater migration and the freshwater stage, while in the former stage the turnover rate of gut microbiome was higher. (3) The gut microbiomes showed higher co-occurrence interactions during freshwater stage than seawater-freshwater transition. (4) “Amino acid metabolism” and “lipid metabolism” were higher during seawater–freshwater transitions than the freshwater stage.

### The Patterns and Drivers of Gut Microbial Diversity During Seawater–Freshwater Migration

Our results found that there was a significant relationship between richness and sampling day of the crab gut microbiome, showing a hump-shaped trend. This is consistent with previous studies that species diversity of gut microbiota changed during migration (Zhang et al., 2020; Lorgen-Ritchie et al., 2021). For example, from freshwater Recirculating Aquaculture System to open marine sea cages, the richness of Atlantic salmon gut microbiome showed a hump-shaped trend (Zhang et al., 2020; Lorgen-Ritchie et al., 2021). In addition, Atlantic salmon reestablished a completely different community structure after freshwater–seawater transition (Lorgen-Ritchie et al., 2021). For bacterial composition, the community dissimilarities increased remarkably over time during migration, and the turnover rate of gut microbiota of the Chinese mitten crab in freshwater was lower than that during seawater–freshwater transition (1–5 days). This difference might be caused by the fact that the water conditions during seawater–freshwater transition changed more dramatically, such as salinity.

Further, we found various variables regulating gut microbiome during seawater-freshwater migration. The key factor affecting alpha diversity and community dissimilarities was the salinity during seawater–freshwater transition (1–5 day). Salinity has frequently been identified as one of the most important environmental factors influencing gut microbiome of aquatic life (Lin et al., 2021) like fish (Lai et al., 2020) and shrimp (Zhang et al., 2016). For example, the proportion of bacteria regarded as opportunists increased while those regarded as commensal or beneficial bacteria decreased when the Nile tilapia and Pacific white shrimp were facing hyposaline or hypersaline stress (Zhang et al., 2016). However, WT and sampling day were the most critical factors in the freshwater stage. The host selection is known as the important force to provide a primary selection of gut microbiota (Xiao et al., 2021). The deterministic process of homogenous selection increased with host development for the assembly of gut microbiota (Dini-Andreote et al., 2015; Burns et al., 2016). For instance, with a 12-day period of studying *Poecilia sphenops*, the fish gut microbiota is mainly driven by host selection independent of water microbiota (Feng et al., 2017).

From the seawater-freshwater transition to the following freshwater environments, the hump-shaped pattern in diversity and the strong turnover of community composition of Chinese mitten crab gut microbiome can be explained by two mechanisms: the intermediate-disturbance hypothesis and the host-selection hypothesis. The intermediate-disturbance hypothesis states that the maximum diversity was caused by the medium frequency or intensity interference or environmental changes (Connell, 1978). Diversity peaks at intermediate disturbance levels because very frequent or intense disturbances eliminate disturbance-intolerant species, while rare or weak disturbances fail to prevent competitive exclusion (Fox, 2013). The drastic changes in salinity may thus lead to an uptick in the diversity of gut microbiome from seawater to freshwater migration. The other hypothesis is that the host development will overwhelm environmental dispersal in governing host gut microbial community succession due to host genetics, immunology, and gut nutrient niches (Xiao et al., 2021). As the migrating host matures, its resistance to further invasion is relatively enhanced. Our study found similar compositions of Chinese mitten crab across regional scales regardless of the ambient environments. Host development may overwhelm environmental proliferation in governing the ecological succession of Chinese mitten crab gut microbiome in the freshwater stage. For instance, the finding on *Poecilia sphenops* suggested that feeding environments have no significant effects on the compositions of fish gut microbiota. Thus, the crab may selectively filter particular microbial members from the environment species pool to function as gut residents at different developmental stages (Yan et al., 2016). Taken together, salinity drives the diversity of gut microbiome of the Chinese mitten crab in seawater to freshwater transition, while host selection would become the dominant factor affecting the diversity of gut microbiome during freshwater stage.

### Temporal Changes in the Gut Microbial Composition

There were differences in the composition of gut microbiome of Chinese mitten crabs during the seawater–freshwater migration. Specifically, Proteobacteria was the dominant abundant phylum by accounting for 86.11% during seawater-freshwater transition (1–5 days), while the relatively abundant phyla were Proteobacteria, Firmicutes, Bacteroidetes, and Actinobacteria in the freshwater stage (Figure 4A). This is consistent with previous studies that the dominant phyla were Bacteroidetes, Proteobacteria, and Firmicutes in the Chinese mitten crabs (Wang et al., 2019). In addition, this discrepancy between seawater and freshwater could be attributed to salinity. For example, previous studies show that the proportion of the bacteria regarded as opportunists increased in the Nile tilapia and Pacific white shrimp upon hyposaline or hypersaline stress, while those regarded as commensal or beneficial bacteria decreased (Zhang et al., 2016).

However, the distributional patterns of bacterial abundance during the seawater–freshwater migration, especially among Chinese mitten crabs, have been rarely explored. For the dominant phylum, the relative abundance of Proteobacteria notably decreased, while Firmicutes significantly increased during the migration. Interestingly, the relative abundance of Bacteroidetes showed a hump-shaped trend during the migration. For instance, the decreasing and increasing patterns of the relative abundance of Proteobacteria and Firmicutes are consistent with the patterns found in previous studies on Atlantic salmon during freshwater–seawater transition (Lorgen-Ritchie et al., 2021). These patterns may be explained by the niche positions of each bacterial phylum. For instance, the niche of Proteobacteria is occupied by other phyla, such as Firmicutes which catabolize complex carbohydrates, polysaccharides, sugars, and fatty acids to provide energy (Tap et al., 2009). Thus, the enrichment of Firmicutes in the gut of Chinese mitten crabs may contribute to energy intake and nutrient absorption (Yang et al., 2016). In addition, Bacteroidetes are highly successful competitors in gut, exhibiting considerable nutritional flexibility and an ability to respond to stresses imposed by the host and the gut environment (Flint and Duncan, 2014). Thus, the change of community compositions in gut microbiome may cause the crab to adapt more quickly during migration.

### Higher Co-occurrence Interactions of Gut Microbiomes in Freshwater Than Seawater–Freshwater Transition

We found that both node-level and network-level topological features of gut microbiome are different between seawater–freshwater transition (1–5 days) and freshwater. For instance, the gut microbiome during seawater–freshwater transition had higher degree value compared to that in freshwater stage. As the topology of the network could reflect interactions between microorganisms, the degree represents the interactions of microbiome in the network (Ma et al., 2016). Our results suggest that the gut microbiome of crab in freshwater had stronger relationships compared to that during seawater–freshwater transition. Meanwhile, the fewer network connections represent the more generalized microbial interactions (Faust and Raes, 2012; Ma et al., 2016; Yang et al., 2021). In addition, the gut microbiome of crab in freshwater had fewer negative co-occurrences than that during seawater–freshwater transition, while negative co-occurrences among taxa may result from the present day or past evolutionary effects of competitive exclusion (Faust et al., 2012; Dohi and Mougi, 2018). It has been proposed that when nutrients are limited, the relationship between microorganisms occupying similar ecological niche can be transformed from commensalism (positive correlations) to competition (negative correlations) (Yue et al., 2019). Thus, the negative association with co-occurrence of the gut microbiome decreased, which may be due to the decreased stocking density of Chinese mitten crabs in the freshwater stage.

### The Shifts of Gut Microbiome in Metabolic Function

“Amino acid metabolism” and “lipid metabolism” had higher contributions during the seawater–freshwater transition (1–5 days), while “carbohydrate metabolism,” “enzyme families,” and “nucleotide metabolism” prominently increased in the freshwater stage. Amino acids, which are major nutrients in the diet, could support the growth of bacteria and their host and also regulate energy and protein homeostasis (Wu, 2009; Dai et al., 2011). Gut Euryhaline mollusks mainly accumulate amino acids as organic osmolytes in their cells under hyperosmotic stress (Pierce and Amende, 2010). For instance, the gut microbiome of snails supports the amino acid supply for the *Theodoxus fluviatilis* production during salinity stress (Kivistik et al., 2020). Thus, the gut microbiome of crab supports the amino acid supply for the host osmolyte production during seawater-freshwater transition. Moreover, lipid metabolism includes the biosynthesis and degradation of lipids such as fatty acids, triglycerides, and cholesterol. Lipid metabolism is mainly regulated by nutrients such as sugars and fatty acids (Schoeler and Caesar, 2019). We speculate that the differences in lipid metabolism and carbohydrate metabolism may be caused by dietary differences.

For phenotypic functions, there are significantly more Gram-negative bacteria and biofilm forming bacteria during seawater–freshwater transition than in freshwater. Such phenotypical differences are mainly caused by the lower proportion of Proteobacteria (Cai et al., 2021; Supplementary Figure 3). Vibrio was Gram-negative Proteobacteria that concerned form biofilms (Shinoda and Miyoshi, 2011), which decreased during seawater–freshwater migration.

## Conclusion

Here, we intensively examined the dynamic changes of diversity, community compositions, co-occurrence network, and potential function of Chinese mitten crab gut microbiome during the seawater–freshwater migration. We found that the species richness of gut microbiome showed a hump-shaped trend over time during the seawater–freshwater migration. Meanwhile, the turnover rate of gut microbiome during seawater–freshwater transition was higher than during freshwater stage. Salinity was the main driver for the species richness and community compositions of gut microbiome of the Chinese mitten crab in seawater-freshwater transition, while host selection may become the dominant factor in the freshwater stage. In addition, gut microbiomes of crab in freshwater have stronger microbial interactions compared to that during seawater–freshwater transition. The changes of metabolism-dependent pathways may assist the host and bacteria themselves to survive in the new environment during migration. Considering that diet and other environmental factors have not been measured, we cannot rule out the effects of other factors on the gut microbiome of Chinese mitten crabs. More studies on host associated systems in the seawater–freshwater transition are needed to verify this concept.

## Data Availability Statement

The datasets presented in this study can be found in online repositories. The names of the repository/repositories and accession number(s) can be found in the article/Supplementary Material.

## Author Contributions

TG conceived and designed the research. TG and CS conducted the sample collection. WZ and CS analyzed the data analyses. CS wrote the manuscript with help from WZ, NL, YL, HZ, JL, ZX, JW, and TG. All authors contributed to the article and approved the submitted version.

## Conflict of Interest

The authors declare that the research was conducted in the absence of any commercial or financial relationships that could be construed as a potential conflict of interest.

## Publisher’s Note

All claims expressed in this article are solely those of the authors and do not necessarily represent those of their affiliated organizations, or those of the publisher, the editors and the reviewers. Any product that may be evaluated in this article, or claim that may be made by its manufacturer, is not guaranteed or endorsed by the publisher.
