# Supplementary material for: Gut Microbiome Succession in Chinese Mitten Crab Eriocheir sinensis During Seawater–Freshwater Migration
Source: Front Microbiol. 2022 Mar 30;13:858508. doi: 10.3389/fmicb.2022.858508 (PMC9005979; doi:10.3389/fmicb.2022.858508)
Supplement: Supplementary file 1 [file Table_1.docx]

**Table S1. Environmental characteristics. WT (**℃**) water temperature. salinity (PSU) water salinity of water. PO4(mg/L) water phosphate. NH3 (mg/L) ammonia nitrogen.**

| SampleID | Group | salinity | NH3 | PO4 | WT |
| --- | --- | --- | --- | --- | --- |
| DJ5G5 | 1D | 20 | 4.2 | 0.23 | 25.1 |
| DJ6G5 | 1D | 20 | 4.1 | 0.21 | 25.2 |
| DJ7G5 | 1D | 20 | 4.4 | 0.23 | 25.8 |
| DJ5G6 | 3D | 15 | 4.3 | 0.25 | 25.3 |
| DJ6G6 | 3D | 15 | 3.8 | 0.21 | 25.4 |
| DJ7G6 | 3D | 15 | 3.8 | 0.22 | 25.5 |
| DJ5G8 | 5D | 10 | 4.2 | 0.27 | 25.8 |
| DJ6G8 | 5D | 10 | 4.4 | 0.24 | 25.4 |
| DJ7G8 | 5D | 10 | 3.6 | 0.29 | 25.3 |
| SH1G2 | 7D | 0 | 2.2 | 0.24 | 26.1 |
| SH2G2 | 7D | 0 | 2.3 | 0.19 | 26.5 |
| SH3G2 | 7D | 0 | 2.1 | 0.18 | 26.8 |
| XH1G2 | 7D | 0 | 2.1 | 0.17 | 26.9 |
| XH2G2 | 7D | 0 | 2.4 | 0.19 | 26.9 |
| XH3G2 | 7D | 0 | 2.3 | 0.16 | 26.9 |
| SH1G3 | 37D | 0 | 2.4 | 0.19 | 27.1 |
| SH2G3 | 37D | 0 | 2.4 | 0.21 | 28.3 |
| SH3G3 | 37D | 0 | 2.3 | 0.21 | 27.4 |
| XH1G3 | 37D | 0 | 2.4 | 0.21 | 26.8 |
| XH2G3 | 37D | 0 | 2.3 | 0.25 | 27.8 |
| XH3G3 | 37D | 0 | 2.2 | 0.24 | 27.8 |
| SH1G4 | 67D | 0 | 2.4 | 0.27 | 28.6 |
| SH2G4 | 67D | 0 | 2.9 | 0.19 | 28.8 |
| SH3G4 | 67D | 0 | 2.4 | 0.23 | 28.6 |
| XH1G4 | 67D | 0 | 2.3 | 0.21 | 28.6 |
| XH2G4 | 67D | 0 | 2.8 | 0.21 | 28.9 |
| XH3G4 | 67D | 0 | 2.4 | 0.23 | 28.4 |
| SH1G5 | 97D | 0 | 2.3 | 0.14 | 29.4 |
| SH2G5 | 97D | 0 | 2.5 | 0.23 | 29.1 |
| SH3G5 | 97D | 0 | 2.7 | 0.17 | 28.8 |
| XH1G5 | 97D | 0 | 2.3 | 0.19 | 29.3 |
| XH2G5 | 97D | 0 | 2.4 | 0.23 | 29.4 |
| XH3G5 | 97D | 0 | 2.3 | 0.21 | 28.9 |
| SH1G6 | 127D | 0 | 2.4 | 0.21 | 25.5 |
| SH2G6 | 127D | 0 | 2.5 | 0.22 | 25.5 |
| SH3G6 | 127D | 0 | 2.8 | 0.24 | 26.5 |
| XH1G6 | 127D | 0 | 3.3 | 0.25 | 27.6 |
| XH2G6 | 127D | 0 | 3.4 | 0.21 | 27.6 |
| XH3G6 | 127D | 0 | 3.1 | 0.19 | 26.6 |
| DJ5W5 | 1DW | 20 | 4.2 | 0.23 | 25.1 |
| DJ6W5 | 1DW | 20 | 4.1 | 0.21 | 25.2 |
| DJ7W5 | 1DW | 20 | 4.4 | 0.23 | 25.8 |
| DJ5W6 | 3DW | 15 | 4.3 | 0.25 | 25.3 |
| DJ6W6 | 3DW | 15 | 3.8 | 0.21 | 25.4 |
| DJ7W6 | 3DW | 15 | 3.8 | 0.22 | 25.5 |
| DJ5W8 | 5DW | 10 | 4.2 | 0.27 | 25.8 |
| DJ6W8 | 5DW | 10 | 4.4 | 0.24 | 25.4 |
| DJ7W8 | 5DW | 10 | 3.6 | 0.29 | 25.3 |
| SH1W2 | 7DW | 0 | 2.1 | 0.18 | 26.8 |
| XH1W2 | 7DW | 0 | 2.1 | 0.17 | 26.9 |
| XH2W2 | 7DW | 0 | 2.4 | 0.19 | 26.9 |
| SH2W3 | 37DW | 0 | 2.4 | 0.19 | 27.1 |
| SH1W3 | 37DW | 0 | 2.4 | 0.21 | 28.3 |
| XH2W3 | 37DW | 0 | 2.3 | 0.21 | 27.4 |
| XH1W3 | 37DW | 0 | 2.4 | 0.21 | 26.8 |
| SH1W4 | 67DW | 0 | 2.9 | 0.19 | 28.8 |
| SH2W4 | 67DW | 0 | 2.4 | 0.23 | 28.6 |
| XH1W4 | 67DW | 0 | 2.3 | 0.21 | 28.6 |
| XH1W5 | 97DW | 0 | 2.3 | 0.14 | 29.4 |
| XH2W5 | 97DW | 0 | 2.5 | 0.23 | 29.1 |
| SH2W5 | 97DW | 0 | 2.7 | 0.17 | 28.8 |
| SH1W5 | 97DW | 0 | 2.3 | 0.19 | 29.3 |
| SH1W6 | 127DW | 0 | 2.5 | 0.22 | 25.5 |
| SH2W6 | 127DW | 0 | 2.8 | 0.24 | 26.5 |
| SH3W6 | 127DW | 0 | 3.3 | 0.25 | 27.6 |
