# Supplementary material for: Gut Microbiome Succession in Chinese Mitten Crab Eriocheir sinensis During Seawater–Freshwater Migration
Source: Front Microbiol. 2022 Mar 30;13:858508. doi: 10.3389/fmicb.2022.858508 (PMC9005979; doi:10.3389/fmicb.2022.858508)
Supplement: Supplementary file 2 [file Data_Sheet_1.docx]

Supplementary Material


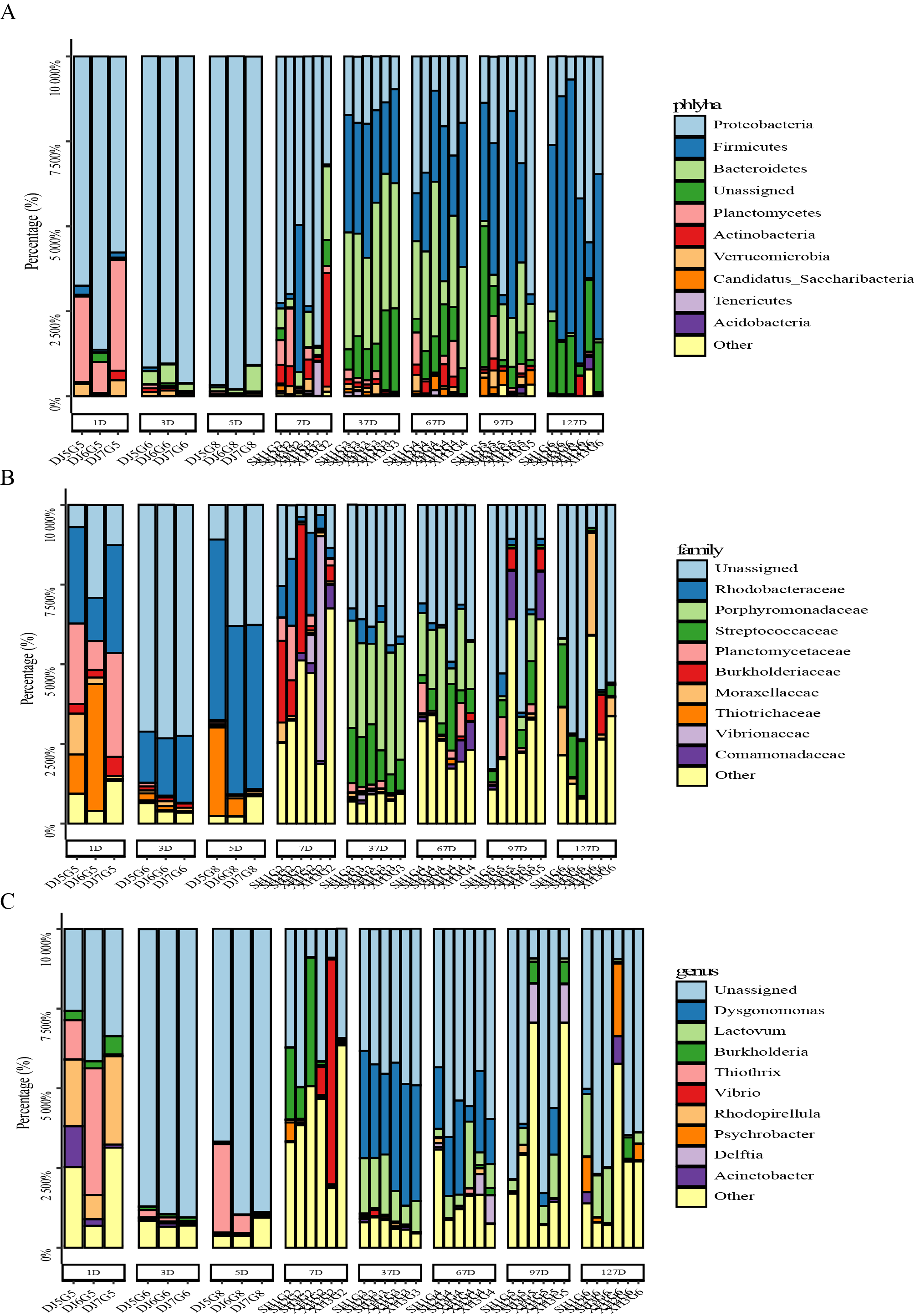


**Supplementary Figure 1.** Stackbars showing the relative abundance of top-10 bacterial **(A)** phyla, **(B)** families and **(C)** genus across all biologically replicated samples. The bacterial taxonomic ranks which have lower relative abundance were grouped into “Other”.


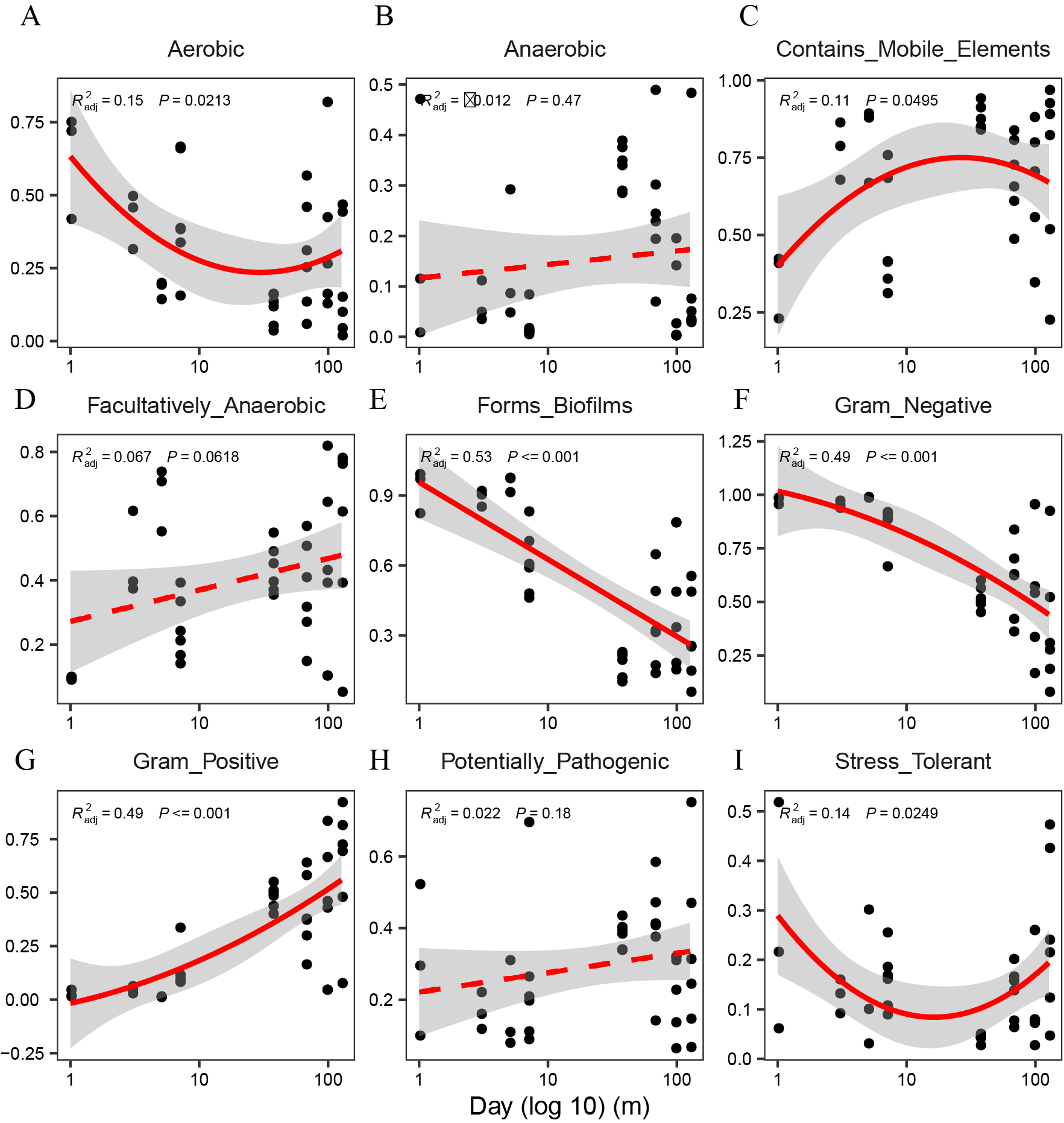


**Supplementary Figure 2** The following organism-level phenotypes were inferred by BugBase. Relative abundances of bacteria differing in Gram staining were shown in (F) for Gram negative and (G) for Gram positive. Relative abundances of bacteria differing in oxygen tolerance phenotypes were shown in (A) for Aerobic, (B) for Anaerobic and (D) for Facultatively Anaerobic. Relative abundances of bacteria differing in latent pathogenicity phenotypes were shown in (C) for containing mobile elements, (H) for potentially pathogenic, (I) for oxidative stress tolerance and (J) for biofilm formation.


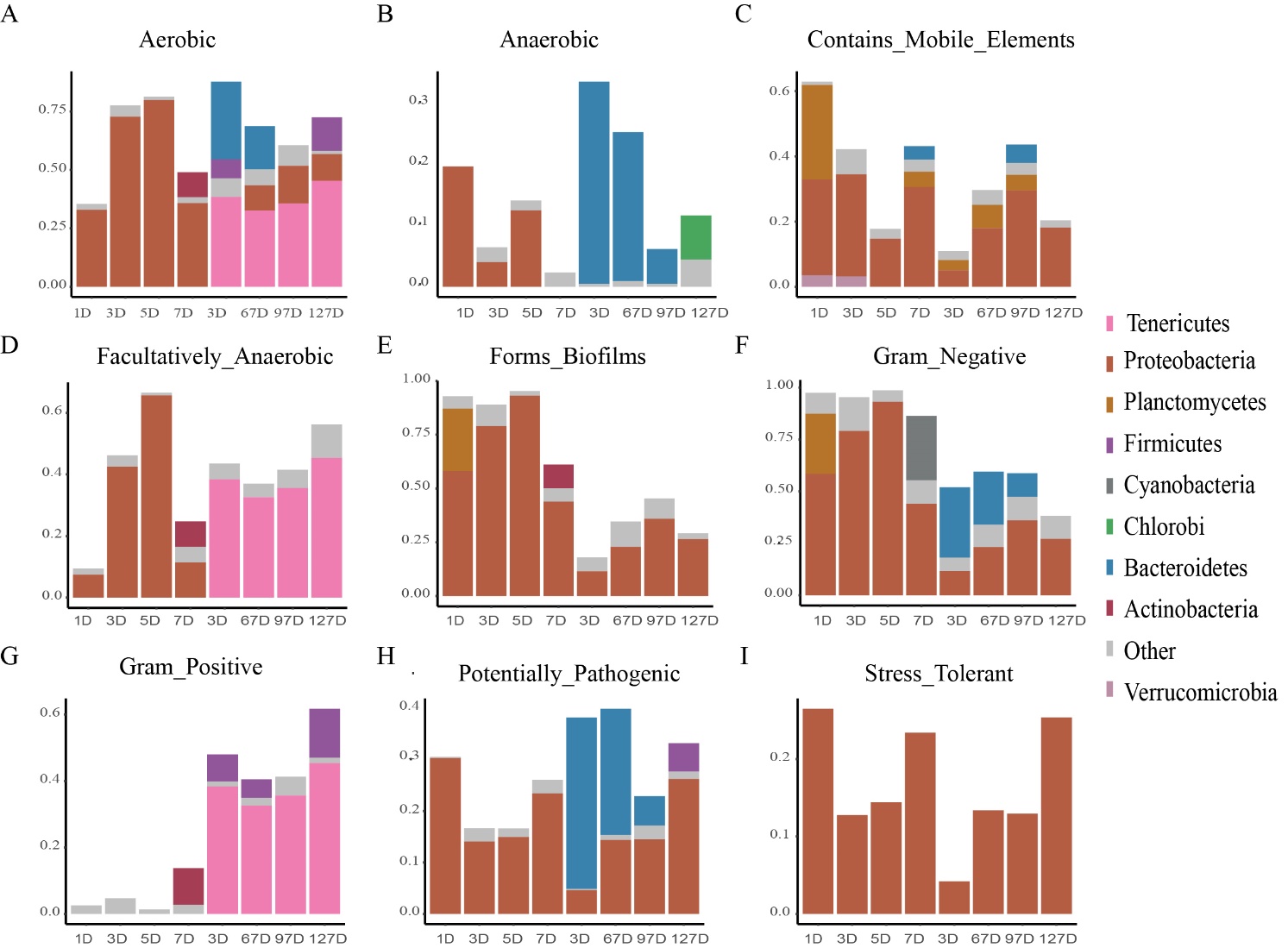


**Supplementary Figure 3** Stackbars showing the relative abundance of bacterial families in contributing to the proportions of bacterial phenotypes that are infered by BugBase. Different sample groups of bacterial communities were shown in x-axis.
